# Positive and Negative Supervisor Development Feedback, Team Harmonious Innovation Passion and Team Creativity

**DOI:** 10.3389/fpsyg.2021.681910

**Published:** 2021-09-02

**Authors:** Yuchun Xiao, Shuwei Liu, Ting Dai

**Affiliations:** ^1^School of Business Administration, Zhejiang Gongshang University, Hangzhou, China; ^2^School of Security Management, Zhejiang College of Security Technology, Wenzhou, China; ^3^Department of Accounting, School of Business, The Hong Kong University of Science and Technology, Kowloon, Hong Kong

**Keywords:** supervisor developmental feedback, team harmony innovation passion, team creativity, proactive personality, innovation

## Abstract

In modern organizations, creative work is usually carried out by teams, and the study of team creativity will therefore have meaningful implications for organization innovation research. The improvement of team creativity is a key management challenge for organization leaders. But our knowledge of how teams respond to and benefit from the supervisor’s developmental feedback is limited. This paper draws on the interdependence and knowledge density of team creativity to study how the supervisor’s developmental feedback influences creativity at the team level. Our statistical analysis of 94 supervisors and 330 employees finds that positive and negative development feedback from the supervisor both have a positive impact on team creativity, the impact from the negative development feedback is even stronger, and we also finds that the team’s harmonious innovation passion mediates the relationship between the supervisor’s developmental feedback and team creativity. In addition, we conclude that proactive personality activity moderates the relationship between the supervisor’s positive (negative) developmental feedback and the team’s harmonious innovation passion. Our research promotes the development of the study of team creativity in the Chinese cultural context and it is also an important application of developmental feedback that can be incorporated into management practices to enhance team creativity.

## Introduction

Creativity enables organizations to innovate and maintain a competitive advantage in a complex and rapidly changing business world. The question of how to improve creativity in an organization preoccupies both industry managers and management scholars. The lack of creativity in some organizations is due to a lack of proper feedback, which creates information asymmetry in the team. Feedback is commonly used to motivate and guide employee’s behavior (Kluger and DeNisi, 1996). Creative research defines “feedback” as information provided by others that helps to judge “creativity” against a relative or absolute standard (Zhou, 1998). The complexity of the creative task often makes employees feel “blind” or unsure about how to work on their job. Employee creativity often occurs in short and unplanned interactions between team members (George and Zhou, 2007). High-quality supervisor feedback can help the team to understand, correct and improve the progress of the creative task in a timely manner (Peng and Chiu, 2010). In Eastern culture, supervisor feedback is viewed as more important by employees than subordinates or colleagues (Ashford and Tsui, 1991), and it is the most commonly used communication method in organizations (Majumdar, 2015). Supervisor Developmental Feedback (SDF) is a typical leadership feedback behavior, which seeks to provide employees with helpful and valuable information that enables them to learn, develop and improve during their work (Zhou, 1998). SDF also encourages employees to improve their own abilities by setting higher goals (Zhou, 2003; Guo and Liao, 2014). Organizations increasingly realize the importance of SDF, especially in comparison to traditional performance feedback methods, and they also apply it widely to contemporary creative tasks.

Feedback, one of the five core work characteristics proposed in the Hackman’s Job Characteristics Model, has been (relatively) insufficiently engaged by Chinese scholars (Guo and Liao, 2014). However, in recent years, SDF has begun to receive attention in China. Some studies discuss the effects of SDF on employees, its effect on knowledge sharing between subordinates (Su and Lin, 2020), involvement in creative work (Hao and Sun, 2020), role in opinion-linked behavior (Liu and Gu, 2018), link to organizational citizenship behavior (Yin and Zheng, 2011), contribution to subordinate evasive behavior by subordinates (Song and Wang, 2015), relation to innovative behavior (Su and Lin, 2018b) and impact on performance (Zheng et al., 2015). But the relationship between SDF and creative work in China background remains under-studied, the only studies are from the paper of Geng et al. (2020) (about the mechanism between SDF and Employee Creativity), the paper of Xu et al. (2018) (regarding Supervisor Developmental Feedback and Creativity), the paper of Wang et al. (2017) (about influence of SDF on Individual Creativity), and the paper of Yin and Zheng (2011) (for the Citizenship Behavior forested by SDF).

The conclusions of above papers are also inconsistent. Some indicate that the relationship between SDF and employee’s creativity is not significant (George and Zhou, 2007), and others suggest they are positively related (Yin and Zheng, 2011; Yanhong et al., 2014; Xu et al., 2018). Other papers also study the relationship between valence of feedback and creativity, such as the discussion about the effect of positive(negative) feedback on team motivation and performance (Van Dijk and Kluger, 2011); the discussion regarding how new or existing negative feedback affect corporate actions and subsequent performance (Eggers and Suh, 2019), and the discussion about the impact of negative feedback on recipient’s creativity (Kim and Kim, 2020), etc. Regarding the relationship between positive feedback (negative feedback) and creativity, scholars’ views are inconsistent as well. Some existing theories and empirical evidence are contradictory. They mainly focus on SDF effects at the individual level and do not sufficiently consider the effect on teams level. We have found one paper studies the relationship between feedback valence and creativity which is applied on a team-centered theoretical perspective (Hoever et al., 2018), but this paper used a random experimental method. Feedback in that paper was given in the ongoing task (experiment). Since the team was organized temporarily, this paper did not study how the feedback valence from a completed task affects the team creativity (TC). Meanwhile, this paper focuses on the regular feedback valence rather than developmental feedback. Therefore, it is necessary for us to further develop the theory of supervisor development feedback valence at the team level.

Team Creativity refers to the ability of team members to generate novel and useful ideas through cooperation (Wang et al., 2016). Both individual and TC focus on the joint novelty and practicality of ideas and solutions (George, 2007). In contrast to individual creativity, the solution of TC consists of multiple interdependent participants (Hoever et al., 2012). Previous research conclusions about the effect of SDF on individual creativity may not be fully applicable to TC. Research into the antecedents of TC addresses various factors, including: (1) team structural factors, such as the cognitive diversity of a team (Li et al., 2012) and gender diversity (Zhang and Zhang, 2012); (2) team processes, such as team conflict (Fairchild and Hunter, 2014) and knowledge-sharing (Zhu et al., 2015); (3) cognition, such as an interactive memory system (Wang and Xue, 2011); collective psychological ownership (Wei et al., 2019); (4) emotional and intrinsic motivation (Tang et al., 2011); and (5) leadership characteristics (Wang and Chen, 2010) and style (Chen et al., 2013). At the global level, there are also relatively few studies of supervisor behavior. Just one paper studies the relationship between SDF and TC (Joo et al., 2012), and there is no study of the distinguishing features of SDF valence and the interactive effects of SDF on TC. In addition, none of these papers refer to the Chinese context.

Chinese “face” culture (Mianzi Culture), which establishes a series of culturally appropriate behaviors and customs that relate to individual status, reinforces the tendency for Chinese supervisors to provide feedback to subordinates through positive means. This raises the important question of how positive and negative SDF affects TC, which strongly depends on interactions among team members. Feedback is an universal management tool. What impact does SDF have on TC? Using SDF to improve TC raises an important question: For positive and negative feedback, which one is more effective (Hoever et al., 2018)? SDF is a type of feedback aiming to enhancing creativity. Our study intends to further verify the different valences of SDF (positive and negative) on the effect TC. We hope to provide a theoretical basis to help manger select a proper feedback method to improve creativity at team level. Hence, we draw on Control Theory and Feedback Intervention Theory (Kluger and DeNisi, 1996) to establish a model of creativity and innovation (Amabile, 1997; Hennessey and Amabile, 1988) as the overall framework. We studied how the positive and the negative feedback which originated from same source predict TC, and which valence (positive or negative) of supervisor development feedback has more impact on TC. We added an explanation of the impact of supervisor developmental feedback on TC from the perspective of valence. This distinction and synchronization verification is very important. In real practice, supervisors used to provide both positive and negative development feedback to subordinates. Meanwhile, we use non-random empirical methods to examine the mechanism and the impact of positive and negative SDF effects on creativity at the team level. We engage a stable team of employees and supervisors and focus on the core role of the Team’s Harmonious Innovation Passion (THIP), which is the highest expression of team motivation (Wei and Zhang, 2018). We address TC, and ask how positive and negative SDF affects the creative outcome for different team characteristics (high and low initiative personality). We seek to: (1) explore if SDF at different valences can all positively affect TC; (2) use THIP to reveal the affects path through which positive and negative SDF influences TC; and (3) adopt a Proactive Personality (PP) perspective to explore the boundary conditions that enable positive and negative SDF to influence TC.

## Theoretical Background and Hypotheses

### Hypothesis

#### Positive/Negative Supervisor Development Feedback (PSDF and NSDF) and Team Creativity (TC)

Supervisor Development Feedback provides employees with valuable information that can be used to effectively improve their capabilities; describe current work and performance and provide benign suggestions that will assist the future development of employees (Zhou, 2003). Employees will be able to develop their potential without restrictions on their freedom of thought and they will not be subject to the will of other people (Zhou, 1998); SDF will enable employees to generate more inspiration and creativity by devoting themselves to creative tasks (Jia et al., 2014) and it will also promote creativity by generating new ideas that can be applied to challenging tasks (An et al., 2020).

Zheng et al. (2015) was the first to divide SDF into positive and negative in accordance with valence, Positive/Negative Supervisor Development Feedback (PSDF and NSDF) are different but related concepts. PSDF means that the individual’s ideas are more creative than the standard, and it is a positive incentive method (which is known as “giving face” in China) that helps employees complete their task to a higher standard of performance (Ma and Yan, 2020). In NSDF, the individual’s ideas are less creative than the standard (Zhou, 1998). The purpose of negative feedback is to correct employees’ bad or ineffective behavior (Zheng et al., 2015). Both positive and negative SDF enable development expectations to be conveyed from supervisors to employees, and they also enable employees to establish individual innovative standards; it also improves their creative capability, including the generation of creative ideas.

As mentioned in the previous section, there is no clear consensus on the relationship between PSDF (NSDF) and creativity. The existing theory and empirical evidence are even contradictory. Some researchers claim that positive feedback benefits employee creativity (Zhou, 1998), others suggest that it inhibits the development of creativity. This also applies to the relationship between negative feedback and creativity, as some scholars point to a positive (Vuori and Huy, 2016) or negative relationship, claim there is no direct effect (George and Zhou, 2001), hold there is no relationship or claim that negative Feedback will hinder creativity (Van Dijk and Kluger, 2011). The literature contains evidence of a positive, negative and null relationship between negative feedback and recipient creativity (Kim and Kim, 2020). Table 1 lists some scholars’ views on the impact of positive feedback and negative feedback on creativity.

**TABLE 1:** Summary of research on PSDF(NSDF) and creativity.

Valence of feedback	Author, years	Direction of effect	Conclusion
PSDF	Zhou, 1998	Positive	Positive feedback is conducive to employee creativity
	George, 2007	Positive	Positive feedback could bring positive common belief in team creativity
	Li et al., 2012	Positive	Subordinates will show the highest level of creativity when they receive positive feedback from the leader
	Vandenberghe et al., 2019	Positive	Positive feedback can strengthen and develop employee in achieving success contentiously
	Ma and Yan, 2020	Negative	Positive feedback is detrimental to creativity. Frequent use of positive supervisor feedback will increase unhealthy self-esteem, strengthen the dependence of employee behavior on external feedback, and guide individuals towards safe and conservative performance goal, which inhibit creativity
PSDF	Fodor and Carver, 2000; George and Zhou, 2001	Null	Negative feedback has no direct effect on the recipient’s creativity
	Ford and Gioia, 2000; Fang et al., 2014; Vuori and Huy, 2016	Positive	Negative feedback is a hard to accept but effective communication tool
	Ilies and Judge, 2005; Van Dijk and Kluger, 2011	Negative	Negative feedback hinders creativity
	Jaussi and Dionne, 2003; Wang and Yan, 2010	Negative	Behaviors such as strict control of employees by supervisors (i.e., negative supervisor development feedback) will inhibit employees creativity
	Kim and Kim, 2020	Positive,Negative, Null	In the bottom-up feedback flow, negative feedback makes employees dissatisfied with the current level of creativity. This dissatisfaction in turn encourages feedback recipients to processes creativity task more carefully In the top-down feedback flow, negative feedback hinders the recipient’s creativity due to the meta-process (the mental state of the negative feedback threat felt by the recipient)

Positive Supervisor Development Feedback means that the individual’s ideas are more creative than the standard required. PSDF is a positive incentive method (which is known as “giving face” in China) which encourage employees do their task with a higher quality standard (Ma and Yan, 2020). NSDF is provided when the individual’s ideas are less creative than the standard required (Zhou, 1998). The purpose of negative feedback is to correct employees’ bad or ineffective behavior (Zheng et al., 2015). Negative feedback has a positive impact on creativity as it makes employees dissatisfied with the current level of creativity and in turn encourages feedback recipients to treat involved creativity tasks more carefully (Kim and Kim, 2020).

Control Theory can effectively explain the purpose of human behavior. The main idea of Control Theory is that when employees see the difference between their actual performance and standard requirements, employees will be motivated to take action (we also call this behavior flow as feedback loop). The job of a supervisor is to promote and strengthen the differences of perception among employees in order to improve team members’ performance. The feedback loop can be either a positive or negative feedback (Stallings, 1974). An example of negative feedback is that supervisor send a signal to employees when their performance is below the standard requirement, the employee will take action to improve his performance until the performance requirement is reached. The positive feedback example is that supervisors used to send a positive signal to employees to encourage them continue outperforming from standards. Individuals would adjust their performance to eliminate the gap between the actual and expected performance from supervisors. Meanwhile, we also suggest future research “to examine the parallel use of positive and negative feedback loops to shape human behavior toward expected performance standards” (Jeffrey A. Miles, 40 must-read theories in organization and management research, M, 2017).

In some creative work, the supervisor deliberately use negative feedback to stimulate TC and avoid employee complacency. This negative feedback may not be the true evaluation to subordinates. Indeed, regardless PSDF or NSDF, SDF reflects the development expectations of employees by conveying information related to their future learning, work, and development to employees. SDF helps to build up employees’ learning and innovation channels, and to reduce risks and uncertainty for new tasks. SDF helps individuals to establish innovation requirement, to increase creative thinking, and to generate creativity. Though both forms of SDF have positively effect on TC, the boundary conditions are different. We therefore predict:

*Hypothesis1a: PSDF positively affects TC*
*Hypothesis1b: NSDF positively affects TC*

#### The Mediating Role of Team Harmony Innovation Passion (THIP)

Harmonious passion is one of the most important driving forces of creativity and innovation (Wei and Zhang, 2018), and it can enhance the level of creativity level (Qin and Zhao, 2015). The harmonious innovation passion is an innovation motivation that the whole team possesses, and it is likely to mediate the relationship between positive (negative) SDF and TC. There are a number of reasons for this:

First, SDF can effectively enhance employees’ intrinsic motivation and help them generate creative ideas and behaviors (Ryan and Deci, 2000). Harmonious passion is the autonomous motivation for SDF that is influenced by contextual factors (Vallerand et al., 2003). These factors can mutually internalize team members’ independent creativity, and can also enhance the team’s harmonious innovation passion. SDF is an autonomous support factor that does not only stimulate employees’ harmonious passions, but also encourages team members to share their identities and internalize each others’ creativity on the basis of positive (negative) SDF. The development feedback generates team identities and forms a harmonious innovation passion at the team level (Wei and Zhang, 2018).

Second, THIP helps to enhance TC. Intrinsic and autonomously formed motivation is the driving force that encourages employees to engage in creative activities (Shin and Zhou, 2007). When the subordinates’ intrinsic work motivation is stimulated, their creativity capability simultaneously improves (Amabile, 1997). Those with intrinsic motivation prefer complexity and novel tasks, seek a higher level of challenge and are more likely to identify alternative solutions (Shin and Zhou, 2007). Motivated team members are more likely to generate new and useful insights (Dugosh and Paulus, 2005). In a team setting, THIP enhances TC and makes employees more willing to share and discuss information and produce novel and useful solutions (van Knippenberg et al., 2004).

Finally, employee motivation plays a very important mediating role in the relationship between supervisor behavior and employee creativity/innovation (George and Zhou, 2007; Hirst et al., 2009). Under appropriate conditions, SDF promotes employee creativity performance by enhancing TC (Amabile, 1983). Appropriate leadership behaviors (such as positive or negative SDF) in a passionate team can make a positive contribution to TC (Wei and Zhang, 2018) and Positive (negative) SDF can increase THIP. And THIP also generates an internal force that leads the team to internalize innovation and develop a shared team identity. They will invest time and energy in innovation and generate novel and useful solutions (Wei and Zhang, 2018) that enhance TC. On this basis, we predict:

*Hypothesis2: THIP will mediate the relationship between PSDF (NSDF) and TC.*

#### The Moderating Effect of Proactive Personality

In considering the influence of feedback recipients on the feedback process, we identify that it is important to study individual differences in feedback recipients (Linderbaum and Levy, 2010). PP is a stable individual difference variable that affects individual proactive behavior, and it has a positive effect on individual innovation behavior. PP is an important predictor of innovation behavior or creativity (Strauss et al., 2015), as employees with high PP are usually highly committed to work goals, and are willing to apply a proactive work attitude to the achievement of these goals (Su and Lin, 2018a). These employees would therefore take NSDF as diagnostic information that helps to correct errors and develop required capabilities. NSDF will not create social embarrassment for these employees and it will not result in threats against them.

Employees with low active personality tend to passively adapt to environmental changes and have an intimate working style. However, they have a weak understanding and acceptance of NSDF, which means that PP is more likely to affect the relationship between PSDF (NSDF) and THIP. We predict that THIP will mediate the impacts of PSDF (NSDF) on TC and also predict that PP will affect TC by changing the THIP driven by PSDF (NSDF). On this basis, we propose the following hypotheses:

*Hypothesis3a*: *For teams with low PP, the impacts of PSDF on TC will be enhanced through the indirect effects of THIP.*

*Hypothesis3b*: *For teams with high PP, the impacts of NSDF on TC will be enhanced through the indirect effects of THIP.*

**FIGURE 1:**
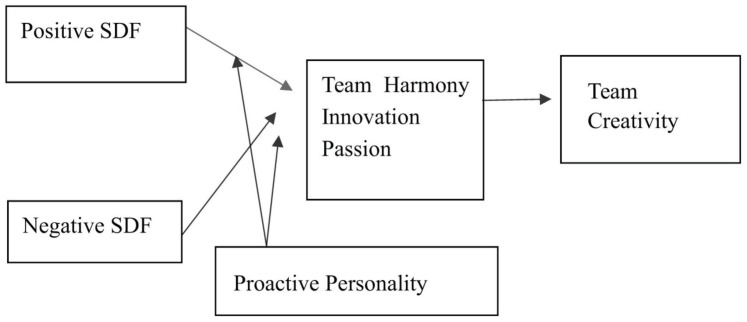
Research model.

On the basis of the above research hypotheses, we construct the research model shown in Figure 1.

### Sample Selection and Data Collection

The data of this research is collected from eight enterprises located in Guangdong, Zhejiang, Jilin and Hebei that work in the energy, finance, manufacturing, medical care, and education industries. We collect data by applying the Supervisors-Employees matching method and conducting a two-part survey. Team supervisors first evaluate TC and the subordinates then complete an evaluation report that refers to SDF (positive and negative), THIP and their personalities. Interviewees also provide their demographic information in the survey. A total of 115 questionnaires are distributed to team supervisors and 397 questionnaires to subordinates. The response rate of effective questionnaires for supervisors is 81.7%, and 83.1% for subordinates. Finally, 424 valid questionnaires are obtained (94 from supervisors and 330 from employees). There are 185 male (56.0%) and 145 female (44%) employees; those aged under 30 years-of-age account for 33.3% and those aged over 30 years-of-age for 66.7%. Three hundred twenty-six employees (70.3%) have an undergraduate degree or above; 108 employees (32.7%) have more than 5 years of working experience and there is an average of 4.51 employees in each team.

### Variable Measurement

Our study mainly uses a Likert 7-point scale for analysis. With the exception of the control variables, all variables are evaluated by this scale, which ranges from 1 (“completely inconsistent”) to 7 (“completely consistent”). A higher value indicates a greater degree of recognition.

Supervisor Developmental Feedback is evaluated by the eight-item scale developed by Zheng et al. (2015). Five items are used for PSDF and three for NSDF. Typical test items include: (1) “When giving feedback, my supervisor recognizes my ability and provides useful information on how to improve my work performance” and (2) “When giving feedback, my supervisor will criticize my weakness by comparing with other colleagues, and would provide me with useful information on how to improve work performance.”

Team Creativity is measured by applying Shin and Zhou’s (2007) four-item scale. Typical test items include “we have a lot of creative ideas.”

Team’s Harmonious Innovation Passion is measured by the scale developed by Vallerand et al. (2003). We ask each team member to evaluate THIP by referring to seven items. Typical test items include: (1) “Innovation gives our team various working experiences.” (2) “Innovation is in harmony with other activities in our team.” (3) “For our team, innovation is a passion that we can manage it.”

Proactive Personality is measured by Seibert et al. (1999)’s scale, which has a total of 10 items. Typical test items include: (1) “I am constantly looking for new ways to improve myself.” and (2) “I will find a way to make a work done if I am believe in no matter hard the work is.”

#### Control Variables

Both demographic variables (Bernerth and Aguinis, 2016), and the average job tenure of a supervisor (Shin and Zhou, 2007) have effect on TC. We also control age, gender, and job tenure of the supervisors same as Vandenberghe et al. (2019). Except for the control variables, other variables were evaluated by using the Likert 7-point scale. 1 means “completely inconsistent” and 7 means “completely in line.” The larger the value, the higher the degree of agreement with the statement.

## Results

Mplus7.0 and SPSS23.0 are used to analyze the data.

### Common Method Bias

Table 2 shows that the five factors (PSDF, NSDF, PP, THIP, and TC) are well aligned with each other in the model (*x^2^* = 622.71; *df* = 367, *x^2^/df* = 1.70; CFI = 0.95; TLI = 0.95; RMSEA = 0.05), and this applies to a greater extent than other nested models. The results show that the variables have good discrimination validity and confirm that the study’s measurements are reliable, well-structured and suited to subsequent data analysis.

**TABLE 2:** Results of confirmatory factor analysis.

Measurement model	*x* ^2^	*df*	*x^2^/df*	CFI	TLI	RMSEA	SRMR	Δχ^2^/Δdf
BI factor model	494.34	339	1.46	0.97	0.97	0.04	0.05	3.96
Five factor model	605.30	367	1.65	0.96	0.95	0.04	0.04	265.76
Four factor model	1668.35	371	4.50	0.77	0.75	0.10	0.10	39.18
Three factor model	1785.88	374	4.78	0.75	0.73	0.11	0.11	534.77
Two factor model	2855.42	376	7.59	0.56	0.52	0.14	0.13	628.05
One factor model	3483.47	377	9.24	0.45	0.40	0.16	0.14	−

We also uses the common method variance (CMV) to test the common method bias. On the basis of the five-factor model (PSDF, NSDF, PP, THIP, and TC), we added another factor which has the common origination of above five factors. The results after adding CMV shows no significant improvement for each fitting index compared with the five-factor model with higher fitting degree, ΔCFI = 0.01, Δ,TFI = 0.02, ΔRMSEA = 0.00, ΔSRMR = 0.01. Therefore, CMV shows that the research model does not have obvious deviation from the common method.

### Reliability and Validity Test

We first perform confirmatory factor analysis (CFA) on the model’s variables. The load of each variable measurement item is between 0.61 and 0.90. They all exceed 0.60, and most are above 0.70. The composite reliability (CR) of PSDF is 0.92; NSDF is 0.86; THIP is 0.91; TC is 0.90; and PP is 0.90. All CR is greater than 0.70. The average extraction variance (AVE) for PSDF is 0.69; NSDF is 0.66; THIP is 0.60; and TC is 0.70. With the exception of the AVE of PP (0.47), the AVE of all other variables exceeds 0.50. Though the AVE of the PP is less than 0.50, PP can still be well measured as the combined confidential degree is acceptable, and the final result is significant. It is undeniable that some data (individual items) collected in our questionnaires interfere with the value of AVE, and bring unsatisfactory effect on our test tool. We would explained more in section “Limitation.” But on the whole, the internal consistency of each variable, the degree of confidence and the aggregation validity are fine and well acceptable.

### Data Aggregation Test

Our research focus on the effect of SDF on creativity at team level. We aggregated individual measurements of team members into team-level variables. Our core variable, TC, is scored by the supervisors directly. Other variables, PSDF, NSDF, THIP, and PP are aggregated to team level. The Rwg (mean) and Rwg (median) of TC, PSDF, NSDF, THIP, and PP are all relatively high and exceed 0.70. Table 3 shows that the ICC1 values of the five variables are all between 0.10 and 0.50, which indicates they have suitable inter-group differences. Four of the variables’ ICC2 exceed 0.70, and one is just below (0.63), which also illustrate they have good inter-group reliability. The values of (Rwg, ICC1, and ICC2) the five variables are ideal and can be aggregated at the team level.

**TABLE 3:** Team level variable aggregation test results.

Variables	Rwg Median	Rwg Average	ICC1	ICC2
Team Creativity	0.89	0.86	0.39	0.70
Positive Supervisor Development Feedback (PSDF)	0.92	0.86	0.41	0.71
Negative Supervisor Development Feedback (NSDF)	0.83	0.78	0.49	0.77
Team Harmony Innovation Passion (THIP)	0.96	0.93	0.47	0.76
Proactive Personality (PP)	0.97	0.89	0.33	0.63

*Five factors model: include PP, PSDF, NSDF, THIP, TC.*

### Variables Statistical Descriptive and Correlation Coefficient Analysis

Table 4 describe the data at team level, which listing mean, standard deviation and correlation coefficient between main variables. Table 4 shows that the correlation coefficients of PSDF, NSDF, THIP, and TC are respectively 0.41 (*P* < 0.01), 0.59 (*P* < 0.01), 0.47 (*P* < 0.01), and 0.61 (P < 0.01). This indicates that PSDF is positively related to THIP and TC and that NSDF is positively correlated with THIP and TC (these correlations are stronger than those related to PSDF). The correlation coefficient between THIP and TC is 0.57 (*P* < 0.01), which indicates that THIP and TC are positively correlated. These results broadly support our hypotheses.

**TABLE 4:** Descriptive analysis and correlation coefficient of variables.

Variables	M	SD	1	2	3	4	5	6	7
1. Age of Supervisors	3.02	1.34							
2. Gender of Supervisors	0.33	0.47	−0.15						
3. Tenure Month of Supervisors in Teams	49.83	50.09	0.30**	0.03					
4. Positive Supervisor Development Feedback (PSDF)	5.10	0.97	0.18	−0.03	0.25*				
5. Negative Supervisor Development Feedback (NSDF)	4.60	1.17	0.02	−0.30**	0.06	0.37**			
6. Proactive Personality (PP)	5.00	0.67	0.09	−0.17	0.06	0.24*	0.49**		
7. Team Harmony Innovation Passion (THIP)	5.08	0.86	−0.02	−0.19	0.11	0.41**	0.59**	0.31**	
8. Team Creativity (TC)	4.84	0.91	0.08	−0.08	0.11	0.47**	0.61**	0.40**	0.57**

(1) Data are described at team level; (2) The age in questionnaire is recorded as a continuous variable with 1–6 grades (1:30 years old; 2:30–35 years old, 3:36–40 years old, 4:41–45 years old, 5:46–50 years old, 6:50 years old and above), the value in the table is the average of six grades; (3) The values in the table are standardized regression coefficients *P < 0.05, **P < 0.01,***P < 0.001.

### Main Effects Test

We adopt the Hierarchical Regression Analysis Method, and the test results are shown in M2 in Table 5. When the regression model is applied, the control variables are not found to be significant. In referring to the control variables, we add PSDF, NSDF, and PP to the regression, and find that the regression coefficient of PSDF on TC is 0.27 (*P* < 0.01), the regression coefficient of NSDF on TC is 0.49 (*P* < 0.001) and ΔR^2^ is statistically significant. The results turn out that the PSDF and NSDF are both positively correlated with TC, but they have different coefficients on the TC. This shows that PSDF has a significant positive impact on TC, and NSDF also has a significant positive impact on TC. These impacts are also stronger than those observed in the case of PSDF. The results support H1a and H1b.

**TABLE 5:** Results of regression analysis.

Variables	Team Creativity (TC)		Team Harmony Innovation Passion (THIP)		
	M1	M2	M3	M4	M5
Constant	4.73***	0.95	5.27***	2.51***	2.21***
**First Step: Control Variables**					
Age of Team Supervisors	0.04	0.02	–0.10	–0.10	–0.05
Gender of Team Supervisors	–0.08	0.09	–0.20	–0.05	–0.06
Tenure Months of Supervisors in Teams	0.10	–0.006	0.14	0.05	0.06
**Second Step: Main Effect**					
PSDF		0.27**		0.23*	0.31**
NSDF		0.49***		0.49***	0.37***
PP		0.11		–0.01	–0.03
**Third Step: Intermediary effect**					
PSDF × PP					−0.24**
NSDF × PP					0.28**
△R^2^	0.02	0.44***	0.06	0.34***	0.16***
F	0.60	12.56	1.77	9.81	10.87

The values in the table are standardized regression coefficients *P < 0.05, **P < 0.01, ***P < 0.001.

The result also shows that NSDF has a stronger effect on TC, which is constant to our hypothesis. By providing NSDF, individual’s ideas are less creative than the standard (Zhou, 1998). After receiving negative feedback, people will make greater adjustments to their next action (Zhu et al., 2019). The purpose of negative feedback is to correct employees’ bad or ineffective behavior (Zheng et al., 2015). Team members who receive negative feedback will search and review the information that not used properly (Hoever et al., 2018). In Kim and Kim’s (2020) paper, negative feedback has a positive impact on creativity as it makes employees dissatisfied with the current creativity, and in turn encourages feedback recipients to carefully study the processes creativity tasks they involved. Therefore, under certain conditions, NSDF has a stronger impact on TC than PSDF.

We also found that negative feedback will bring more motivation for team as well as pressures. The negative impact on the individual’s emotions and experience from negative feedback is obvious and straightforward, but the impact on creativity, especially on TC, is complex. The impact of NSDF on TC is not as simple as generally considered. Although the effect on TC in our research is positive, it may only be an intermediate impact at a certain stage. Frequent negative feedback could bring great psychological pressure to team members, and thus negatively affect creativity. But as team members could encourage each other when facing psychological pressure, the diversified characteristics and information process methods from team members may help team members turn pressure into motivation, and bring positive effects on TC. We believe the effect of NSDF is even stronger than PSDP.

### Mediating Effects

We use *Process software* to repeatedly sample 5,000 times, in accordance with the Self-Repetitive Sampling Method (see Table 6). THIP mediates the relationship between PSDF and TC (Boot 95 percent CI = [0.1158,0.3085]) and the relationship between NSDF and TC (Boot 95 percent CI ([0.0974,0.6521]); THIP has a significant impact on TC (b (=0.26, Boot 95 percent CI = [0.0790, 0.4170]; PSDF has a significant positive effect on TC (b = 0.21, Boot 95 percent CI = [0.0060,0.4150]); NSDF has a significant positive impact on TC (b = 0.41, Boot 95 percent CI = [0.2240, 0.6000]); And this coefficient is stronger than the one from PSDF. THIP also has a partial mediating effect on the relationship between PSDF (NSDF) and TC. Hypothesis 2 is therefore verified.

**TABLE 6:** Results of mediation effect analysis.

Control Variables	Team Harmony Innovation Passion (THIP)			Team Creativity (TC)		
	M6			M7		
	b	SE	Boot 95% CI	b	SE	Boot 95% CI
**Control Variables**						
Age of Team Supervisors	−0.07	0.63	[−0.199, 0.049]	0.04	0.07	[−0.099,0.178]
Gender of Team Supervisors	−0.10	0.22	[−0.538, 0.317]	0.22	0.16	[−0.098,0.515]
Tenure Months of Supervisors in Teams	0.000	0.002	[−0.002,0.004]	0.00	0.00	[−0.004,0.004]
**Independent Variables**						
PSDF	0.23	0.10	[0.051,0.462]	0.21	0.11	[0.006,0.415]
NSDF	0.49	0.12	[0.254,0.711]	0.41	0.10	[0.224, 0.600]
**Mediating Variable**						
THIP				0.26	0.09	[0.079, 0.417]
R^2^		0.40***			0.49***	
				SE		Boot 95% CI
Intermediary effect	THIP		0.06	0.05		[0.1158,0.3085]
			0.13	0.05		[0.0974, 0.6521]

The values in the table are standardized regression coefficients *P < 0.05, **P < 0.01, ***P < 0.001.

**FIGURE 2:**
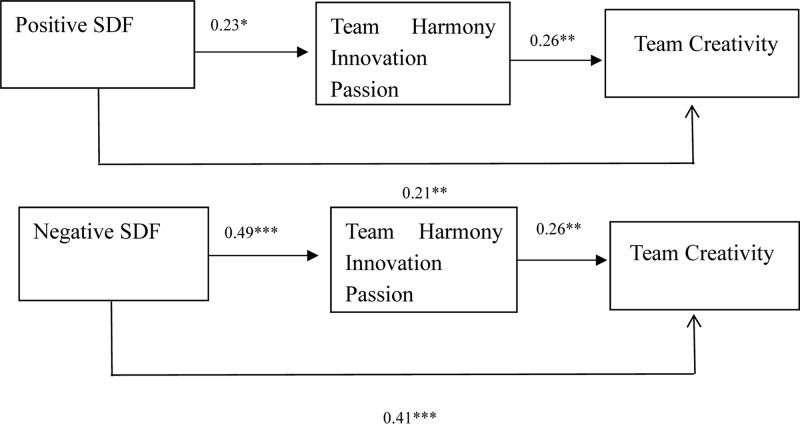
Conditional direct and indirect effects for PSDF and NSDF. The values in the table are standardized regression coefficients.

In order to more clearly explain the relationship and influence of PSDF (NSDF) on the intermediary variables (the direct effect), THIP, and TC (the indirect effect), we draw Figure 2. Figure 2 can clearly show the PSDF has less impact on THIP and TC than NSDF does.

### Adjusting Effect Test

**FIGURE 3:**
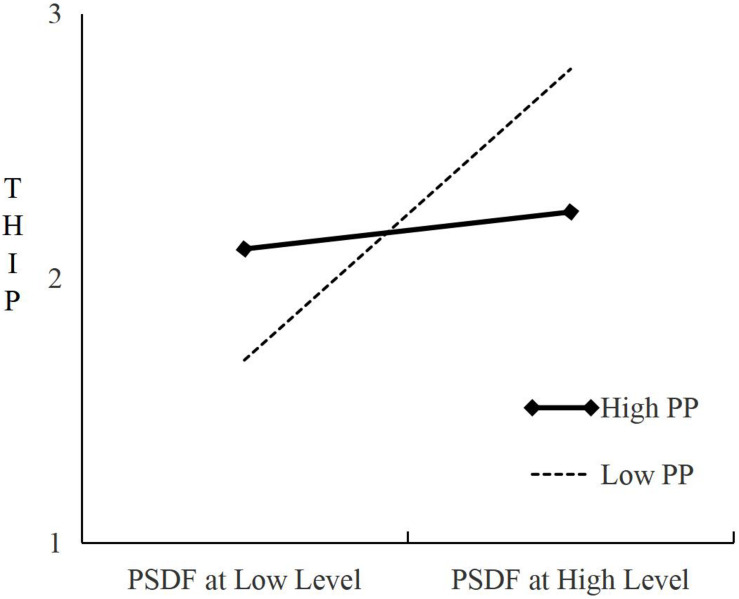
Moderating effect of proactive personality under PSDF.

**FIGURE 4:**
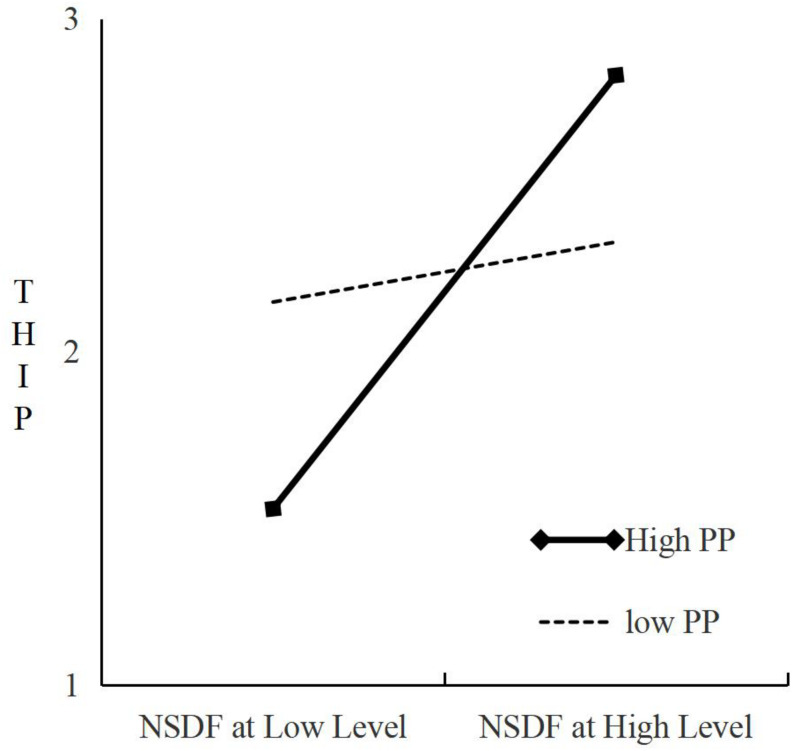
Moderating effect of proactive personality under NSDF.

We seek to test the moderating effect of the PP on the indirect relationship of “PSDF (NSDF) → THIP→ TC.” Interaction variable (PSDF (NSDF) × PP) is added on the basis of the main effect test. Refer to Table 4, where M4 shows that PSDF has a significant positive impact on THIP (b = 0.23, *P* < 0.05 and ΔR^2^ is statistically significant); NSDF has a significant positive impact on the THIP (b = 0.49, *P* < 0.001 and ΔR^2^ is statistically significant). Again refer to Table 4, where M5 shows the Interaction variable of PSDF and PP has a positive effect on THIP: the regression coefficient is −0.24; *P* < 0.01 and ΔR^2^ is statistically significant. This indicates that PP has a significant negative adjustment effect on the relationship between PSDF and THIP. The regression coefficient of the interaction variable of NSDF and PP on THIP is 0.28, *P* < 0.01 and ΔR^2^ is statistically significant. The relationship between SDF and THIP has a significant positive adjustment effect on TC. Refer to Figure 3, where the Simple Slope test shows that low initiative personality has *t* = 4.60 (*P* < 0.001) and high initiative personality has *t* = 1.35 (*P* = 0.18, not significant), which supports the hypothesis that PP has a partial moderating effect on the relationship between PSDF and THIP. H3a is therefore partially established. Refer to Figure 4, where the Simple Slope test shows that low initiative personality has *t* = 2.38 (*P* < 0.05) and high initiative personality has *t* = 6.29 (*P* <0.01). This supports the claim that PP has a moderating effect on the relationship between NSDF and the THPFI. H3b is therefore verified.

## Main Research Conclusion and Contributions

### Conclusions

Both positive and negative SDF positively impact TC. NSDF has a stronger impact on TC than PSDF. When NSDF is matched and there is high PP in a team, TC will be enhanced through THIP.

Second, THIP has a partial mediating effect on the relationship between PSDF (NSDF) and TC. In order for THIP to be generated, intervention must first occur through PSDF (NSDF). The stimulation of PSDF (NSDF) enables THIP to optimize, and TC is enhanced as a result.

Third, PP has a moderating effect on the relationship between NSDF and THPFI. A team with high PP can influence the THIP by interacting with NSDF, and it then indirectly affects TC. Teams with low PP can influence the THIP by interacting with PSDF, and then indirectly affect TC.

### Theoretical Contribution

Academia has been increasingly interested in the formation and mechanism of organizational organizational behavior variables in the context of Eastern management in recent years (Chuang et al., 2015). In line with this, our paper studies the developmental feedback mechanisms PSDF and NSDF in China, and we contribute to the literature in four key aspects:

First, we adopt a development feedback perspective to explore the effects of supervisor leadership on TC. By conducting empirical research into the PSDF and NSDF variables (which originate from one source), we provide further insight into how SDF influences TC at the valence angle, and which valence has a stronger effect on TC. We thereby explain the impact of SDF on TC from the perspective of valence. This distinction and synchronization verification is critical, because in practice supervisors are likely to provide both PSDF and NSDF to subordinates simultaneously. In this way our research fills the gap left by previous studies, which lack a discussion of valence, and provides a theoretical basis on which supervisors can select better strategies to improve TC.

Second, unlike previous studies, which focus on the individual, our research extends the relationship between SDF and creativity to teams. Teams have meaning and dynamic characteristics that cannot be explained or covered by individual data. Our research presents the impact of the interaction between motivation and process on innovation at the team level. We believe it is an important test, and an extension of Amabile’s creativity and innovation element model. Since the advent of Amabile’s model, empirical research has focused mostly on individual-level research. However, research at the individual level ignores the interdependence and knowledge-intensive nature of TC. Our paper reveals the complex mechanism of PSDF (NSDF) on TC from the perspective of motivation, and finds that even NSDF at the team level can increase TC. This paper enriches the literature on the relationship between SDF and TC, expands the discussion of the SDF model itself, verifies the role of PP in the SDF model, and extends the research boundary to the influence of SDF on TC.

Third, we reveal the complex mechanism of PSDF and NSDF on TC from a motivation perspective, and specifically answer the question “Why do positive and negative SDF lead to different levels of TC respectively?” In our answer, we provide a new perspective from which to explain SDF’s effectiveness. Either positive or negative SDF can enhance collective identity among team members, generate a higher THIP, increase knowledge sharing and communication between team members, and stimulate TC. The difference between PSDF and NSDF is their effectiveness on TC. We provide empirical support to further clarify the obscure relationship between SDF and TC, and enrich the literature on THIP field. Our paper provides a theoretical basis from which managers can select motivation strategies for team members when the team’s creativity is failing.

Fourth, our paper draws from Feedback Intervention Theory (Kluger and DeNisi, 1996) to establish an empirical model of creativity and innovation (Amabile, 1997; Hennessey and Amabile, 1988) as its overall framework. Our paper then applies Zheng et al.’s (2015) PSDF and NSDF Measurement Table to the empirical results. Although other scholars have studied the relationship between team-centered feedback valence and creativity (Hoever et al., 2018), their research mainly adopted the random experimental method (in which the experiment is conducted in a temporary organization environment) and they did not gain sufficient data from the subsequent tasks (Hoever et al., 2018). Our study uses a non-random empirical method, in which the research object is a stable team of employees and supervisors. We tested different SDFs on the team, and the method and impact of positive and negative SDF on TC. This experiment gives managers a theoretical base that can guide their feedback practices.

### Practical Contribution

We provide a new analytical mode that managers can incorporate into a valance perspective, which can then be used to improve TC. Our developmental feedback can be used to improve creativity, and we provide valuable suggestions that PSDF (NSDF) can use to stimulate TC. We also show that organizations should seek to enhance TC by strengthening supervisors’ ability to provide developmental feedback.

Second, we show how THIP can be used to inspire employees. The inspiring of passion “from the heart” is a subject that has been long discussed in management practice. We provide a solution and show how supervisors can use PSDF and NSDF to stimulate THIP, and ultimately enhance TC. When teams have low levels of enthusiasm, more SDF stimuli (positive or negative) can help to generate more harmonious passion, and this will ultimately promote TC.

Third, we show managers should take proactive personality characteristics into account when considering the kind of developmental feedback they provide to the team. When teams have high PP, more NSDF can be used to stimulate THIP, as employees can generate a desire for innovation, and this can ultimately enhance TC. But when teams have low PP, we suggest that managers should instead use PSDF to stimulate harmonious innovation passion.

### Research Limitations and Prospects

Due to the time and resources constrains, our study still has some improvement space. First, our data sample are small and lack vast representative in region and industry. Future research should expand the sample size and collect data from multiple industries to make sample more diversified. Second, creativity judgment is inherently subjective and self-conscious, which is difficult to assess. Although we have used two measurement methods, self-evaluation and other-evaluation, there still has bias in the measurement of TC due to subjective reasons. Thirdly, our research used questionnaire survey to collect cross-sectional data. This data may not fully reflect the longitudinal causal relationship between variables. A longitudinal and time-point research is needed. Fourth, matching supervisor and employees’ data can avoid common method bias, but the only using of questionnaire survey method is insufficient. We would suggest future researchers to combine questionnaires and experimental method together to further explore the factors that can affect TC from PSDF and NSDF. Fifth, The collection of control variables is not sufficient in our survey. Future research should take more control variables into consideration. The measurement of PP (AVE) is lower than 0.50, and some individual level items on the questionnaire interfere with the value of AVE. Future research should test the tools and select measurement that are more in line with the sample. Lastly, our paper explores the influence of motivation mechanism from SDF to TC. In the future, we can try to explore the influence of other intermediary mechanisms such as affection and team conflict.

## Data Availability Statement

The original contributions presented in the study are included in the article/Supplementary Material, further inquiries can be directed to the corresponding author.

## Author Contributions

All authors listed have made a substantial, direct and intellectual contribution to the work, and approved it for publication.

## Conflict of Interest

The authors declare that the research was conducted in the absence of any commercial or financial relationships that could be construed as a potential conflict of interest.

## Publisher’s Note

All claims expressed in this article are solely those of the authors and do not necessarily represent those of their affiliated organizations, or those of the publisher, the editors and the reviewers. Any product that may be evaluated in this article, or claim that may be made by its manufacturer, is not guaranteed or endorsed by the publisher.
