# Supplementary material for: Positive and Negative Supervisor Development Feedback, Team Harmonious Innovation Passion and Team Creativity
Source: Front Psychol. 2021 Sep 2;12:681910. doi: 10.3389/fpsyg.2021.681910 (PMC8444987; doi:10.3389/fpsyg.2021.681910)
Supplement: Supplementary file 1 [file Data_Sheet_1.pdf]

APPENDIX A

Positive Supervisor Development Feedback(PSDF)

(1)My supervisor uses expressions of approval or praise when providing feedback to improve my job performance.

(2)My supervisor offers developmental feedback based on his or her approval of my work results.

(3)My supervisor inspires me to think how to accomplish tasks more efficiently through praising some of my work behaviors or tactics.

(4)When providing feedback ,my supervisor recognizes my skills for task completion and helps me improve .

(5)When giving me feedback,my supervisor recognizes my competence compared with other employees and provides me with useful information on how to improve my job performance.

Negative Supervisor Development Feedback(NSDF)

(1)My supervisor uses negative expressions or criticism to give feedback when providing feedback to improve my job performance.

(2)My supervisor inspires me to think how to accomplish tasks more efficiently through criticizing some of my work behaviors or tactics.

(3)When giving me feedback,my supervisor criticizes my lack of competence compared with other employees and provides me with useful information on how to improve my job performance.

Proactive Personality（PP）

(1)I am constantly on the lookout for new ways to improve my life.

(2)Wherever I have been,I have been a powerful force for constructive change.

(3) Nothing is more exciting than seeing my ideas turn into reality.

(4)If I see something I don’t like, I fix it.

(5)No matter what the odds,if I believe in something I will make it happen.

(6)I love being a champion for my ideas,even against others’ opposition.

(7)I excel at identifying opportunities.

(8)I am always looking for better ways to do things.

(9) If I believe in an idea.no obstacle will prevent me from making it happen.

(10)I can spot a good opportunities long before others can.

Team Harmony Innovation Passion（THIP）

(1)This activity allows me to live a variety of experiences.

(2)The new things that I discover with this activity allow me to appreciate it even more.

(3)This activity allows me to live memorable experiences.

(4)This activity reflects the qualities I like about myself .

(5) This activity is in harmony with the other activities in my life.

(6)For me it is a passion,that I still manage to control.

(7)I am completely taken with this activity.

Team Creativity（TC）

(1)The team always comes up with idea of newness .

(2)The team always comes up with idea of usefulness.

(3)The team is creative.

(4)The new ideas proposed by the team are significance to the company.
