# Supplementary material for: Positive and Negative Supervisor Development Feedback, Team Harmonious Innovation Passion and Team Creativity
Source: Front Psychol. 2021 Sep 2;12:681910. doi: 10.3389/fpsyg.2021.681910 (PMC8444987; doi:10.3389/fpsyg.2021.681910)
Supplement: Supplementary file 2 [file Data_Sheet_2.pdf]

Appendix I

Team Overall Evaluation - Employee Section

Thank you for filling out the questionnaire. All the information you filled in will be completely confidential. All data is for academic research and analysis only. Please fill in base on your actual feelings and opinions. Thank you very much for your support and participation!

School of Business Administration, Zhejiang Gongshang University

	Please mark the number (1-7) that best fits the actual situation in your team, (1 means "Completely Inconsistent", 7 means "Completely in Line"):	Completely Inconsistent			Generally Consistent		Completely in line	
1	My supervisor uses expressions of approval or praise when providing feedback to improve my job performance.	1	2	3	4	5	6	7
2	My supervisor offers developmental feedback based on his or her approval of my work results.	1	2	3	4	5	6	7
3	My supervisor inspires me to think how to accomplish tasks more efficiently through praising some of my work behaviors or tactics.	1	2	3	4	5	6	7
4	When providing feedback ,my supervisor recognizes my skills for task completion and helps me improve .	1	2	3	4	5	6	7
5	When giving me feedback,my supervisor recognizes my competence compared with other employees and provides me with useful information on how to improve my job performance.	1	2	3	4	5	6	7
6	My supervisor uses negative expressions or criticism to give feedback when providing feedback to improve my job performance.	1	2	3	4	5	6	7

7	My supervisor inspires me to think how to accomplish tasks more efficiently through criticizing some of my work behaviors or tactics.	1	2	3	4	5	6	7
8	When giving me feedback,my supervisor criticizes my lack of competence compared with other employees and provides me with useful information on how to improve my job performance.	1	2	3	4	5	6	7
9	I think I am good at coming up with new ideas.	1	2	3	4	5	6	7
10	I am confident in my ability to solve problems creatively.	1	2	3	4	5	6	7
11	I have the ability to develop new ideas based on the ideas of others.	1	2	3	4	5	6	7
Please mark the number (1-7) that best fits the actual situation in your team, (1 means "Completely Inconsistent", 7 means "Completely in Line"):		Completely Inconsistent		Generally Consistent		Completely in line		
12	I always share professional skills with my teammates	1	2	3	4	5	6	7
13	We always help each other when someones' work behind the schedule	1	2	3	4	5	6	7
14	We always cheer each other up and encourage each other when team is in depressed.	1	2	3	4	5	6	7
15	The feedback from my teammates is very useful	1	2	3	4	5	6	7
16	Teammates can provide me with valuable suggestion to improve my performance	1	2	3	4	5	6	7
17	I always discuss and solve work problems with my teammates	1	2	3	4	5	6	7
18	There often has some one plays as peacemakers in my team when in disputes.	1	2	3	4	5	6	7
19	I am constantly on the lookout for new ways to improve my	1	2	3	4	5	6	7

	life.							
20	Wherever I have been,I have been a powerful force for constructive change.	1	2	3	4	5	6	7
21	Nothing is more exciting than seeing my ideas turn into reality.	1	2	3	4	5	6	7
22	If I see something I don't like, I fix it.	1	2	3	4	5	6	7
23	No matter what the odds,if I believe in something I will make it happen.	1	2	3	4	5	6	7
24	I love being a champion for my ideas,even against others' opposition.	1	2	3	4	5	6	7
25	I excel at identifying opportunities.	1	2	3	4	5	6	7
26	I am always looking for better ways to do things.	1	2	3	4	5	6	7
27	If I believe in an idea.no obstacle will prevent me from making it happen.	1	2	3	4	5	6	7
28	I can spot a good opportunities long before others can.	1	2	3	4	5	6	7
29	This activity allows me to live a variety of experiences.	1	2	3	4	5	6	7
30	The new things that I discover with this activity allow me to appreciate it even more.	1	2	3	4	5	6	7
31	This activity allows me to live memorable experiences.	1	2	3	4	5	6	7
32	This activity reflects the qualities I like about myself .	1	2	3	4	5	6	7
33	This activity is in harmony with the other activities in my life.	1	2	3	4	5	6	7
34	For me it is a passion,that I still manage to control.	1	2	3	4	5	6	7
35	I am completely taken with this activity.	1	2	3	4	5	6	7

Please fill in your personal information according to the actual situation:

1. Gender: ______(1) Male (2) Female

2. Age: _______years old (1) under 30 years old; (2) 30-35 years old; (3) 36-40 years old; (4) 41-45 years old; (5) 46-50 years old; (6) over 50 years old

3. Highest educational background:_______ (1) High school or below; (2) Junior college; (3) Undergraduate; (4) Postgraduate or above

4. Majors studied in school (subject to the major of the highest degree):______ (1) Science and Engineering (Science, Engineering, Agronomy and Medicine) (2) Economics and Management (Economics, Management); (3) Literature and Art (Philosophy, Literature, History); (4) Law; (5) Others (pedagogy and military science and those without education majors)

5. Your tenure in the current team: ________(1) 1 year or below; (2) 1-2 years; (3) 2-3 years; (4) 3-5 years; (5) 5 years or above.

6. The team you work for has a total ________members;

7. You have been working with your directly leader for _________months.

8. Please rate the familiarity between the members of the team.

Completely unfamiliar, General Familiarity, Very familiar with each other

1 2 3 4 5

Thank you very much for your support to our research. Please encapsulate the questionnaire in the original letter and return it to your questionnaire issuer after completing the questionnaire. I wish you success in your work and a happy life!

员工调查问卷

您好！感谢您在百忙之中填写问卷。您所填写的所有内容将完全保密，所有数据仅供学术研究分析使用，恳请根据自己的实际感受和看法如实填写，非常感谢您的支持与参与！

浙江工商大学工商管理学院

请根据您所在团队的实际情况，在最符合实际情况的数字(1-7）划√（1 表示“完全不符”，7 表示“完全符合”)：		完全不符		一般符合			完全符合	
1	我的领导在给我提供反馈以提高工作绩效时，会使用赞许性或表扬性的表达方式	1	2	3	4	5	6	7
2	我的领导基于对我工作结果的认同给我提供发展性反馈	1	2	3	4	5	6	7
3	我的领导会通过赞赏我的一些工作行为或工作技巧来启发我思考如何更高效地完成任务	1	2	3	4	5	6	7
4	在给我反馈时，我的领导认可我在完成工作时所使用的技能，并帮助我改进	1	2	3	4	5	6	7
5	在给我反馈时，我的领导认可我相比其他同事具有的能力，并提供关于如何提高我的工作绩效的有用信息	1	2	3	4	5	6	7
6	我的领导在给我提供反馈以提高工作绩效时，会使用否定性或批评的表达方式	1	2	3	4	5	6	7
7	我的领导会通过批评我的一些工作行为或工作技巧来启发我思考如何更有成效地完成任务	1	2	3	4	5	6	7
8	在给我反馈时，我的领导会批评我相比其他同事欠缺的	1	2	3	4	5	6	7

	能力，并提供给我有关如何提高工作绩效的有用信息							
9	我觉得自己擅长想出新的点子和想法	1	2	3	4	5	6	7
10	工作中，我对自己创造性地解决问题的能力很自信	1	2	3	4	5	6	7
11	我有能力在别人想法的基础上发展新的创意	1	2	3	4	5	6	7
请根据您所在团队的实际情况，在最符合实际情况的数字（1-7）划√（1 表示“完全不符”，7 表示“完全符合”）:		完全不符		一般符合		完全符合		
12	我与部门同事之间彼此分享专业技能	1	2	3	4	5	6	7
13	当部门同事工作进度落后时彼此之间会相互帮忙	1	2	3	4	5	6	7
14	在同事心情低落时，会相互打气鼓励	1	2	3	4	5	6	7
15	我觉得部门同事给我的反馈十分有用	1	2	3	4	5	6	7
16	同事能够提供给我有价值的信息，帮助我改善工作表现	1	2	3	4	5	6	7
17	我与部门同事间共同讨论并解决工作问题	1	2	3	4	5	6	7
18	在纷争产生时，同事间往往会有人出面来当和事佬	1	2	3	4	5	6	7
19	我会经常留心能够改善自己生活的新方式	1	2	3	4	5	6	7
20	无论在哪里，我都是推动建设性发展的力量	1	2	3	4	5	6	7
21	没有什么能比看到自己的想法变成现实更令人激动的事情	1	2	3	4	5	6	7
22	如果看到我不喜欢的事情，我就会改变它	1	2	3	4	5	6	7
23	不管成功的几率有多大，只要我相信，我就会去做	1	2	3	4	5	6	7
24	我会坚持捍卫我的观点，即使有人反对	1	2	3	4	5	6	7
25	我善于识别新的机会	1	2	3	4	5	6	7
26	我总会寻找更好的方法处理事情	1	2	3	4	5	6	7
27	如果我坚信某个想法，任何困难都不能阻止我去实现它	1	2	3	4	5	6	7
28	我能比别人更早地发现好机会	1	2	3	4	5	6	7
29	创新使我们团队有了各种各样的体验	1	2	3	4	5	6	7
30	创新使我们团队有了新发现，使我们更加热爱创新	1	2	3	4	5	6	7

31	创新使我们团队有了难忘的经历	1	2	3	4	5	6	7
32	创新体现了我们团队喜欢的自身所具有的那些特性	1	2	3	4	5	6	7
33	创新与我们团队中的其他活动是和谐的	1	2	3	4	5	6	7
34	对我们团队来说，创新是一种激情，但我们能够掌控它	1	2	3	4	5	6	7
35	我们团队着迷于创新活动	1	2	3	4	5	6	7

请根据实际情况填写您的个人资料：

1.性别：_______(1) 男 (2) 女

2.年龄：______岁 (1) 30 岁以下；(2) 30-35 岁；(3) 36-40 岁；(4) 41-45 岁；(5) 46-50 岁；(6) 50 岁以上

3.最高学历：______ (1) 高中及以下；(2) 大专；(3) 本科；(4) 研究生及以上

4.在校所学专业（以最高学历所学专业为准）：______ (1) 科学工程（理学、工学、农学和医学）(2) 经济管理（经济学、管理学）；(3) 文学艺术（哲学、文学、历史学）；(4) 法律；(5) 其他（教育学和军事学以及无教育专业者）

5.您在目前团队的任职年限：_____ (1) 1 年及以下；(2) 1-2 年；(3) 2-3 年；(4) 3-5 年；(5) 5 年及以上。

6.您工作的团队共有_____个成员；

7.您与直系领导的共事时间有____个月。

8.请您评价一下团队内成员间的熟悉程度

完全不熟悉		熟悉程度一般		彼此非常熟悉
1	2	3	4	5

非常感谢您对本研究的支持；问卷完成后，请将问卷按原信封装好，交还您的问卷发放人。

祝您工作顺利，生活愉快！
