# Supplementary material for: Positive and Negative Supervisor Development Feedback, Team Harmonious Innovation Passion and Team Creativity
Source: Front Psychol. 2021 Sep 2;12:681910. doi: 10.3389/fpsyg.2021.681910 (PMC8444987; doi:10.3389/fpsyg.2021.681910)
Supplement: Supplementary file 3 [file Data_Sheet_3.pdf]

Appendix II

## Performance Evaluation to Individual Employees from Leader

Thank you for filling out the questionnaire. All the information you filled in will be completely confidential. All data is for academic research and analysis only. Please fill in base on your actual feelings and opinions. Thank you very much for your support and participation!

School of Business Administration, Zhejiang Gongshang University

| Please mark the number (1-7) that best fits the actual situation in your team, (1 means "Completely Inconsistent", 7 means "Completely in Line"): | | Completely Inconsistent | | Generally Consistent | | Completely in line | | |
|---|---|---|---|---|---|---|---|---|
| 1 | This employee always comes up with idea of newness . | 1 | 2 | 3 | 4 | 5 | 6 | 7 |
| 2 | This employee always comes up with idea of usefulness. | 1 | 2 | 3 | 4 | 5 | 6 | 7 |
| 3 | This employee is creative. | 1 | 2 | 3 | 4 | 5 | 6 | 7 |
| 4 | The new ideas proposed by this employee are significance to the company. | 1 | 2 | 3 | 4 | 5 | 6 | 7 |
| 5 | This employee always comes up with new ideas to improve the work | 1 | 2 | 3 | 4 | 5 | 6 | 7 |
| 6 | This employee is able to explore new methods, techniques or use new tools at work | 1 | 2 | 3 | 4 | 5 | 6 | 7 |
| 7 | This employee can find original solutions to problems at work | 1 | 2 | 3 | 4 | 5 | 6 | 7 |
| 8 | This employee would do everything possible to | 1 | 2 | 3 | 4 | 5 | 6 | 7 |

| | | | | | | | | |
|---|---|---|---|---|---|---|---|---|
| | support innovative ideas | | | | | | | |
| 9 | This employee is able to find a way to obtain approval for innovative ideas | 1 | 2 | 3 | 4 | 5 | 6 | 7 |
| 10 | This employee can inspire the enthusiasm of important members of the organization for innovative ideas | 1 | 2 | 3 | 4 | 5 | 6 | 7 |
| 11 | This employee is able to transform novel ideas into innovative practices | 1 | 2 | 3 | 4 | 5 | 6 | 7 |
| 12 | This employee can systematically introduce innovative ideas into the work environment | 1 | 2 | 3 | 4 | 5 | 6 | 7 |
| 13 | This employee can correctly evaluate the utility of the innovative idea | 1 | 2 | 3 | 4 | 5 | 6 | 7 |

Thank you very much for your support to our research. Please encapsulate the questionnaire in the original letter and return it to your questionnaire issuer after completing the questionnaire. I wish you success in your work and a happy life!

# 调查问卷（领导问卷）——对团队下属分别评价

您好！感谢您在百忙之中填写问卷。您所填写的所有内容将完全保密，所有数据仅供学术研究分析使用，恳请根据自己的实际感受和看法如实填写，非常感谢您的支持与参与！

| 指导语：以下描述是否适合该下属，请根据发生频次在合适的数字上 (1-7) 划√ (1 表示“完全不符”, 7 表示“完全符合”)： | | 完全不符 | | 一般符合 | | | 完全符合 | |
|---|---|---|---|---|---|---|---|---|
| 1 | 该员工在工作中，会率先尝试新想法或新方法 | 1 | 2 | 3 | 4 | 5 | 6 | 7 |
| 2 | 该员工在工作中，会寻求新方法或新途径来解决问题 | 1 | 2 | 3 | 4 | 5 | 6 | 7 |
| 3 | 该员工在工作中，会产生与工作领域相关的开拓性想法 | 1 | 2 | 3 | 4 | 5 | 6 | 7 |
| 4 | 在员工在工作中，是一个非常优秀的创造力典范 | 1 | 2 | 3 | 4 | 5 | 6 | 7 |
| 5 | 该员工能够提出新想法以改进工作 | 1 | 2 | 3 | 4 | 5 | 6 | 7 |
| 6 | 该员工在工作中能够探索新的方法、技巧或使用新工具 | 1 | 2 | 3 | 4 | 5 | 6 | 7 |
| 7 | 该员工能为工作中的难题找到具有原创性的解决方案 | 1 | 2 | 3 | 4 | 5 | 6 | 7 |
| 8 | 该员工能够想尽办法争取对创新想法的支持 | 1 | 2 | 3 | 4 | 5 | 6 | 7 |
| 9 | 该员工能够想办法获得对创新想法的批准 | 1 | 2 | 3 | 4 | 5 | 6 | 7 |
| 10 | 该员工能够激发组织重要成员对创新想法的热情 | 1 | 2 | 3 | 4 | 5 | 6 | 7 |
| 11 | 该员工能够将新颖的想法转化为创新实践 | 1 | 2 | 3 | 4 | 5 | 6 | 7 |
| 12 | 该员工能够将创新想法系统地引入到工作环境中 | 1 | 2 | 3 | 4 | 5 | 6 | 7 |
| 13 | 该员工能够正确评价创新想法的效用 | 1 | 2 | 3 | 4 | 5 | 6 | 7 |

浙江工商大学工商管理学院

**非常感谢您对本研究的支持；问卷完成后，请将问卷按原信封装好，交还您的问卷发放人。**

**祝您工作顺利，生活愉快！**
